# Hand Gesture Recognition Using EGaIn-Silicone Soft Sensors

**DOI:** 10.3390/s21093204

**Published:** 2021-05-05

**Authors:** Sungtae Shin, Han Ul Yoon, Byungseok Yoo

**Affiliations:** 1 Department of Mechanical Engineering, Dong-A University, Busan 49315, Korea; stshin@dau.ac.kr; 2 Department of Mechanical Engineering, University of Maryland, College Park, MD 20742, USA; 3 Division of Computer and Telecommunication Engineering, Yonsei University, Wonju 26493, Korea; 4 Department of Aerospace Engineering, University of Maryland, College Park, MD 20742, USA

**Keywords:** machine learning, classification, wearable device, soft sensor, hand gesture recognition, silicone strain sensor, eutectic gallium-indium (EGaIn)

## Abstract

Exploiting hand gestures for non-verbal communication has extraordinary potential in HCI. A data glove is an apparatus widely used to recognize hand gestures. To improve the functionality of the data glove, a highly stretchable and reliable signal-to-noise ratio sensor is indispensable. To do this, the study focused on the development of soft silicone microchannel sensors using a Eutectic Gallium-Indium (EGaIn) liquid metal alloy and a hand gesture recognition system via the proposed data glove using the soft sensor. The EGaIn-silicone sensor was uniquely designed to include two sensing channels to monitor the finger joint movements and to facilitate the EGaIn alloy injection into the meander-type microchannels. We recruited 15 participants to collect hand gesture dataset investigating 12 static hand gestures. The dataset was exploited to estimate the performance of the proposed data glove in hand gesture recognition. Additionally, six traditional classification algorithms were studied. From the results, a random forest shows the highest classification accuracy of 97.3% and a linear discriminant analysis shows the lowest accuracy of 87.4%. The non-linearity of the proposed sensor deteriorated the accuracy of LDA, however, the other classifiers adequately overcame it and performed high accuracies (>90%).

## 1. Introduction

Nobody knows the exact origin of gestures in human society, when/how/why we started to use gestures [1]. However, it is definitely true that the usability of gestures as non-verbal communication is limitless. As an example using gestures for communication, sign language is a typical example of the non-verbal communication using hand and finger gestures. Several studies for hand gesture recognitions and sign languages have been reported in the literature. (refer to the review article [2] for more details). Moreover, the hand gesture recognition has been widely used for various applications: not only the sign languages, but also drone control [3], robot arm manipulation [4], and virtual/augmented reality interface [5].

There are several approaches to recognize hand gestures: vision based [6,7,8], data glove based [9,10], and bio-signal based [11,12,13,14]. In particular, this study has focused on hand gesture recognition based on the data glove approach. In the studies using data gloves for the hand gesture recognition, a key topic is manufacturing a high stretchable and reliable signal-to-noise ratio (SNR) sensor which is embedded into a glove to capture hand motions [10,15,16,17,18,19,20,21,22]. For a soft and stretchable sensor, a Eutectic Gallium-Indium (EGaIn) alloy [23] has been a popular choice as a conductive liquid material to be embedded in a silicone matrix to construct microchannels due to its liquid state at room temperature, high surface tension, and outstanding electrical conductance [20]. The EGaIn alloy was utilized for various engineering applications in soft robotics, wearable electronics, stretchable electronics and sensors, and tunable antennae [18,24,25,26]. Other liquid conductors such as carbon greases, ionic liquids, and biocompatible conductive liquids [15,16,19,21] have been investigated to develop soft strain and force sensors. However, since the conductivity of the liquid materials is very low compared to the liquid metal alloy, the associated sensing system requires additional signal conditioning circuitries to acquire a reasonable SNR signal and reliable measurements.

In the surveys of Rashid and Hasan [27] and Chen et al. [8], they introduced four type of sensors for developing data gloves: flex sensor [28,29,30,31], stretch sensor [15,32,33,34,35,36,37], inertial measurement unit (IMU) [38,39], and magnetic sensor [40,41]. IMU sensor is popularly used for measuring the orientation in 3D space by fusing the information of accelerometers, gyroscopes, and magnetometers. Magnetic (or hall-effect) sensor detects the movements in a magnetic field. These IMU and magnetic sensors are popular measurement units for the body movement, however they consist of rigid components that are relatively bulky for a wearable device. Another type of sensor, a flex (or bend) sensor, tends to measure deflection angles. It is widely used for commercial products such as CyberGlove III and 5DT data gloves because of its practical advantages such as along-life cycle and low price. The other sensor type, a stretch (or strain) sensor, is slightly similar to the flex sensor, however, the stretch sensor has a particular characteristic, highly flexibility, which is suitable for human body movements. The proposed sensor in this study can be categorized into the stretch sensor.

The recent works about the stretch sensor for developing data gloves have studied various design concepts and evaluated the performance of their designs. Bianchi et al. [32] used knitted piezo-resistive fabrics to recognize hand postures and to detect tactile pressure as well. Michaud et al. [33] developed a gallium-based soft, resistive and thin (<50 µm) strain sensor to measure finger joint angles, but it was insensitive to normal pressure. Atalay et al. [35] proposed a data glove via the batch manufacturing technology which was easy for the mass production. They developed a data glove with highly stretchable textile-silicone capacitive sensors. Their capacitive sensor had the advantages of high linearity and low hysteresis. Additionally, Ryu et al. [36] devised a knitted glove sensing system with fabric structure strain sensors and evaluated the electrical responses of the tensile and compressive behaviors of the strain sensor. Yeo et al. [42] proposed a strain sensor, which had 150 mm in length and 2 mm in width; and was fabricated by the screen-printing method of silver ink on a thin layer. Their strain sensor was developed to be integrated into a pneumatic soft actuator for building a sensorized actuating glove which could be used for rehabilitation and virtual reality applications.

There were a soft sensor using a conductive liquid metal, EGaIn, similar to our study. Park et al. [34] proposed a soft sensor which consisted of soft silicone, Ecoflex, and a conductive liquid metal, EGaln. The proposed sensor had high elasticity, light weight and reasonable sensitivity. They designed two separated regions to detect the movement of finger joints. Kim et al. [43] introduced a novel fabrication process based on direct ink writing (DIW) of EGaIn in a 2D plane. This fabrication process directly created the whole sensor system for a data glove: the sensing elements, electrical wires, and electrode parts. Nassour et al. [44] also introduced a soft sensor which was fabricated by injecting the conductive liquid metal, EGaIn, into a silicone tube. Based on the soft sensor, they prototyped a data glove to identify 15 hand gestures.

Compared to these previous studies, this study presents the following as the main advantages of the developed EGaIn-silicone microchannel soft sensor.

- Integration of multiple sensory points to detect multiple joints of a finger; generally, hand gestures involving finger movements require two separate sensors to measure the degree of bending (DoB) of a finger.
- High stretchable characteristic; essentially, the proposed soft sensor has higher stretchability and robustness which are came from the material properties of the soft silicone compared to the piezoelectric film which is widely used for sensors of a data glove in the hand gesture recognition applications.
- The possibility to measure dual properties: pressure and strain; fortunately, the proposed EGaIn-silicone microchannel sensor has abilities reacting to being pressured and to being stretched.

In this study, we present the development of a hand gesture monitoring glove based on the soft and robust EGaIn-silicone microchannel sensors to measure finger joint movements. We evaluated the performance of the data glove to 12 hand gestures which are stemmed from American Sign Language. The performance of the data glove was evaluated from the hand gesture dataset collected from 15 human participants. The major contributions of this study are as follows.

- This study developed a EGaIn-silicone based soft sensor for creating a data glove. The proposed sensor was designed for (1) the integration of multiple sensory points; (2) highly stretchable characteristic; and (3) possibility to measure dual properties. Generally, hand gestures related to finger movements require at least two separated sensory points to measure DoB of a finger. In the consideration of the integrated sensor design, it may become easier to install the sensors on a data glove. Consequently, the complexity and the defective rate of the manufacturing procedure of the data glove may decrease.
- The performance of the proposed soft sensor (or the data glove) was evaluated in a real application as the classification of hand gestures. We collected the dataset of the hand gestures from the human subjects and evaluated the performance of the data glove upon six traditional classification algorithms. As interpreting the results, we discussed the functionality of the proposed sensor in the hand gesture recognition.

This paper is organized as follows. Section 2 presents our manufacture procedure to build EGaIn-silicone soft sensors and a data glove for hand gesture recognition; data acquisition of hand gestures; and data analysis used in this work. Section 3 presents results, which are discussed and interpreted in the Discussion (Section 4). Section 5 provides conclusion and possible follow-up work.

## 2. Materials and Methods

### 2.1. EGaIn-Silicone Sensor Fabrication Process

The soft EGaIn-silicone sensor consists of a conductive liquid metal, silicone matrix embedding microchannels, and electrical wires. As a conductive liquid material for filling microchannels, a well-known 99.99% EGaIn alloy (from Sigma-Aldrich, Inc.) [45] with the resistivity of ρ = 29.8 × 10^−8^ Ω/m [23] was used. A platinum-catalyzed Ecoflex 00-30 silicone rubber (from Smooth-on, Inc.) was chosen to be used as a sensor matrix to construct highly deformable microchannels due to its low Young’s modulus (70 kPa), low viscosity (3000 cps), and 900% elongation rate [45].

**Figure 1:**
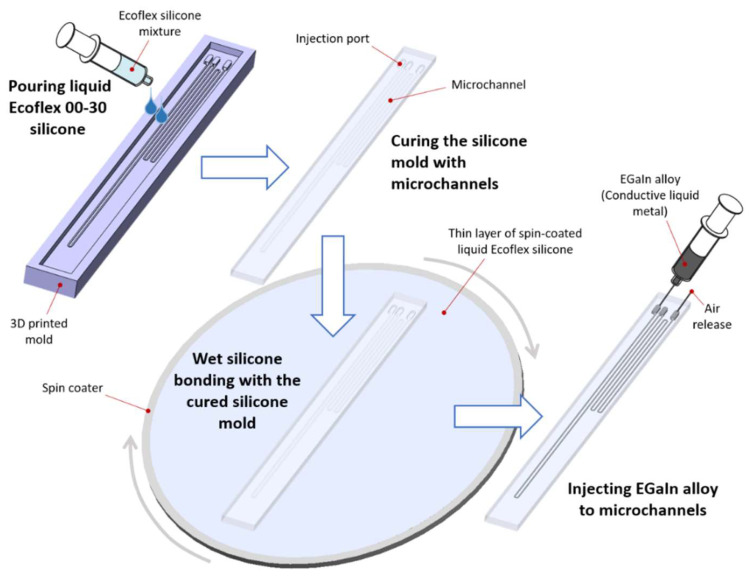
Fabrication process of the EGaIn-silicone sensor.

The fabrication process of the sensor is shown in Figure 1. A plastic mold was prepared using an Object500 Connex2 3D printer (from Stratasys, Ltd.) with a high quality resolution of 16 µm [46] and rigid and durable Vero family photopolymer inks. The mold contained two microchannels based on the design of cross-sectional dimensions of 250(*w*) × 125(*t*) µm. The spacing between microchannels was 500 µm, and the total length of the individual microchannels was set to 160 mm. A liquid Ecoflex 00-30 silicone mixed with two parts of the silicone material was poured into the mold and cured at room temperature for 24 h. A thin silicone layer was prepared by spin-coating the liquid Ecoflex 00-30 silicone. The cured silicone mold with open microchannels was bonded to the spin-coated silicone layer and fully cured at room temperature without additional pressure or heat. Since the spin-coated silicone layer was very thin, we did not experience any microchannel blockage that could result from the wet silicone flowing into the microchannels. A EGaIn alloy was injected into the microchannels while air was released through an additional syringe needle inserted into the other terminal port. Finally, 32 AWG cooper wires were connected to the EGaIn microchannel sensors by inserting them into the terminal ports.

**Figure 2:**
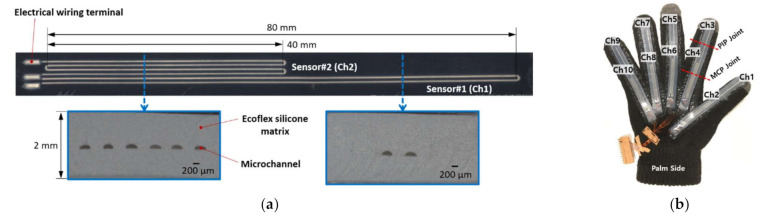
EGaIn-silicone sensor prototypes: (**a**) a EGaIn-silicone sensor and the selected cross-sectional areas of the sensor; (**b**) a hand gesture sensor using a fabric glove installed with the EGaIn-silicone sensors.

A prototype of the EGaIn-silicone sensor with an overall thickness of 2 mm is shown in Figure 2a. The cross section of the microchannel is originally designed in a rectangular shape with a size of 250 (w) × 125 (t) µm. However, as shown in Figure 2a, the final microchannels constructed in the silicone matrix was similar to the crescent moon shape different from the original channel design due to the printing limitations of the polyjet 3D printer using the photopolymer inks and layer-by-layer UV light curing process. As shown in Figure 2a, the microchannel had three electrical wiring terminals designed with approximate dimensions of 2.5 (l) × 1 (w) × 0.625 (t) mm to minimize their resistance changes due to applied strain and to facilitate the electrical wire insertion. The middle terminal was used to provide a 5 V DC input voltage. The total length of both microchannels is equal to 160 mm; therefore, the individual channels are designed to have two different active sensing lengths of 40 (by four folds) and 80 mm (by two folds), respectively, to distinguish the bending positions of finger joints.

A hand gesture sensor system of Figure 2b was fabricated by mounting five EGaIn-silicone sensors on the folds of each finger of the data glove. The five EGaIn-silicone sensors were bonded to the palm side of the data glove using the Ecoflex silicone as an adhesive material. The sensors were decided to be installed on the palm side of the glove, since the change in resistance caused by the folding of the sensor is much more effective than the change by the stretching of the sensor. When the proximal interphalangeal (PIP) joint is bent, a microchannel sensor (Ch1 in Figure 2a) with the active sensing length of 80 mm measures the associated resistance change. On the other hand, the resistance values of both microchannel sensors (Ch1 and Ch2 sensors) vary by the bending of the Metacarpo-phalangeal (MCP) joints. Under ideal finger bending conditions, the number of microchannels in the Ch2 sensor is twice that of the Ch1 sensor, so the change in resistance of the Ch2 sensor should be twice as large as the change in resistance of the Ch1 sensor.

### 2.2. Resistance Change Measurment

**Figure 3:**
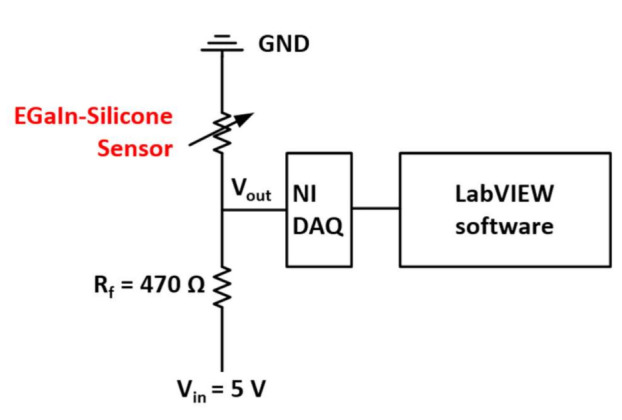
Electronic circuit diagram for measuring the resistance change of the EGaIn-silicone sensor due to the geometrical deformation of the microchannel.

The baseline resistances of the EGaIn-silicone sensor without any deformations were 1.68 Ω and 1.58 Ω for Ch1 and Ch2 sensors, respectively. The resistance values were measured by using the middle terminal as a common ground. The baseline resistance of the two microchannel sensors should be the same because of the microchannels filled with a similar amount of an EGaIn alloy proportional to the same total length. However, we observed that the 3D printed microchannels have some non-uniformity, which could make the difference in resistance measurements. Figure 3 shows the simple voltage divider circuit diagram to read signals from the EGaIn-silicone sensor. The 5 V DC input voltage was continuously supplied by the NI SCB-68 module connected to the NI USB-6251 data acquisition device that measures the voltage difference of the EGaIn-silicone sensor due to sensor deformation.

**Figure 4:**
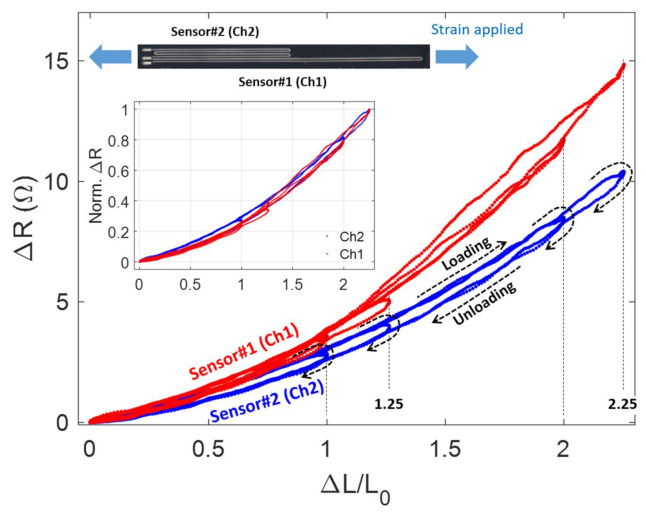
Measurement of resistance change of the EGaIn-silicone sensor while uniaxial strain is applied along the longitudinal direction of the sensor.

Figure 4 shows the change in resistance as long as uniaxial strain is applied along the longitudinal direction of the EGaIn-silicone sensor. The sensor was gradually stretched multiple times at a relatively low speed less than 3 mm/s, with various strain levels of 100, 125, 200, and 225%. The resistance of the EGaIn sensor progressively increases as the sensor is expanded along its length, showing a similar nonlinear profile with measurements. In Figure 4, we observed that the reading data from the Ch1 sensor shows a slightly large nonlinearity than the Ch2 sensor. The Ch1 sensor appears to increase in nonlinearity pattern as the maximum strain level increases, while the Ch2 sensor has a constant nonlinearity. In general, polymer-based sensors have some degree of hysteresis [20]. The Ch2 sensor shows a certain amount of hysteresis; however, the hysteresis of the Ch1 sensor depends on the maximum strain value. For example, the hysteresis level of the Ch1 sensor was much greater for the 225% applied strain compared to the 100% strain case, but the amount of hysteresis was dramatically reduced at about 100% strain level. This hysteresis problem with EGaIn-silicone sensors is likely to be more apparent in pressure sensing applications in a high pressure range of over 40 kPa [20,47]. The hysteresis characteristics of EGaIn-silicone sensors also depends on the strain rate. The level of the hysteresis decreases with increasing strain or pressure rate [20]. The strain sensing results of the Ch2 sensor show the repeatability under the loading and unloading conditions of the sensor, regardless of the level of the strain applied.

### 2.3. Data Acqusition of Hand Gestures

**Figure 5:**
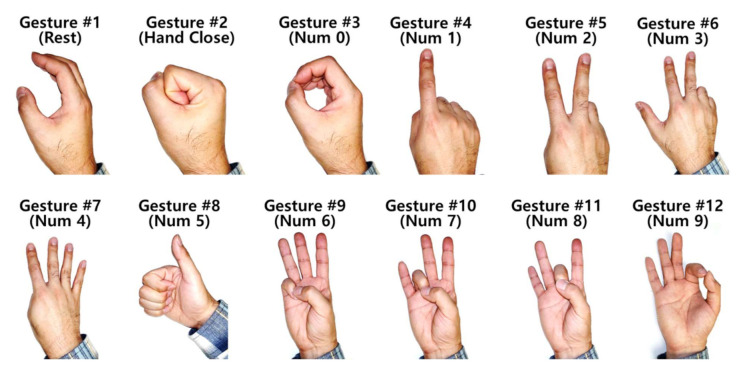
The collected 12 static hand gestures.

In the study, 12 static hand gestures were investigated to estimate the performance, in the hand gesture recognition, of the data glove which the proposed soft (force/stretch) sensors were installed. The investigated hand gestures are shown in Figure 5. We recruited 15 healthy volunteers under the approval of the Institutional Review Board (IRB) at the University of Maryland and written informed consent. We collected the age, gender, height, and weight of the volunteers for the demographic and ethnic information of the study participants shown in Table 1.

The data glove included the 5 proposed soft sensors which have 10 channels in total as shown in Figure 2b and voltages of the channels were measured via the circuit explained in Figure 3. The NI USB-6251 acquired voltage data from the 10 channels of the data glove at 1 kHz sampling rate. The experiment collecting the data glove data of the 12 hand gestures had one type of section. The section was repeated six times for data acquisition. The protocol of the section is as follows:

1. Start at Gesture #1 (Rest)
2. Hold the Gesture #1 for 7 s
3. Return to the Gesture #1 for rest
4. Prepare Gesture #2 (Hand Close)
5. Perform Gesture #2
6. Hold the Gesture #2 for 7 s
7. Return to the Gesture #1 for rest
8. Repeat 4. ~ 7. for Gesture #3 ~ #11
9. Prepare Gesture #12 (*Num 9*)
10. Perform Gesture #12
11. Hold the Gesture #12 for 7 s
12. Return to the Gesture #1 for rest
13. Take a rest for a while (one section has been finished.)

Before starting the experiment, a plenty of practicing time was allowed for a participant to become accustomed to performing the hand gestures and the experiment protocol. After confirming the familiarity from the participant, the experiment and data acquisition was performed.

### 2.4. Analysis of Hand Gesture Recognition

To analyzing the performance of the hand gesture recognition via the proposed data glove, five procedures were performed: (1) preprocessing, (2) segmentation [48], (3) feature extraction, (4) training classifiers, and (5) performance evaluation.

**Figure 6:**
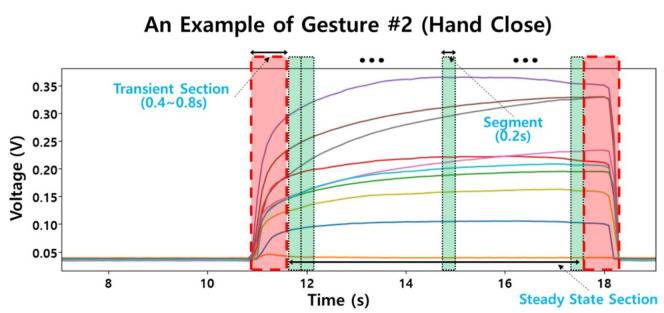
Preprocessing and segmentation for the feature extraction of hand gestures. This figure shows measured voltages of 10 channels of a Gesture #2 (hand close). The start and end transient sections (red regions of 0.4–0.8 s length) are removed at the preprocessing procedure. After the preprocessing, the feature is calculated as the mean value of a window (a green region of 0.2 s length) at the steady state section.

(1) The preprocessing procedure included 2 tasks: removing the start and end transient sections from the analysis and segmenting the steady state section with a prefixed window length to extract features. The length of the transient section is about 0.4 s(s) to 0.8 s; mainly, this was caused from a motion transaction from Gesture #1 (Rest) to a specific gestures and from a specific gesture to Gesture #1 for the rest. The transient section, which was found by the visual inspection of a trained inspector, was removed from the analysis. The investigator inspected the voltage signals of 10 channels visually; (1) finding the onset and end of each gesture trial, and (2) determining the transient sections which was about 0.4–0.8 s long after the onset and before the end as shown in Figure 6.
(2) For the segmentation, the steady state section was windowed by 200 milliseconds (ms) length with no overlapped area. The window length, 200 ms, was empirically selected for a reasonable accuracy and train/test sample size by trial-and-error [48].
(3) For the feature extraction, the mean value of each segment which included 200 samples due to 200 ms of the window length at 1 kHz sampling rate was calculated as a feature. For example, a hand gesture which had a 5 s steady state section had 25 segments per each channel, therefore, this gesture generated a 25 × 10 feature matrix which had 25 samples of 10 feature dimensions from 10 channels of the sensors. Due to this averaging method, no filtering techniques were applied to the raw voltage data in the preprocessing procedure. The total sample size generated from 15 subjects was 36,323. The dataset included 10 feature columns which had floating-point numbers in voltage (V). The total number of classes was 12 stemmed from the static hand gestures investigated in this study.
(4) We chose six traditional machine learning techniques: K-Nearest Neighbors (KNN) [49], Support Vector Machine (SVM) [50], Linear Discriminant Analysis (LDA) [51], Quadratic Discriminant Analysis (QDA) [51], Random Forest (RF) [52], and Naïve Bayes (NB) [53]. We investigated the six traditional classifiers because the results needed to be interpreted by speculating the reasons from the behaviors of the classifiers in the white box (interpretable model) manner. That is why we did not include black-box classifiers such as ANN [50] and Deep learning architectures [54] in this study. The model parameters of the classifiers were estimated by the grid search. The estimated parametres were: KNN: ‘k’ = 2 where ‘k’ is the number of neighbors; LDA: ‘n_components’ = 1 where ‘n_components’ is the number of components; QDA: ‘reg_param’ = 0.001 where ‘reg_param’ is the regularization of the per-class covariance; SVM: ‘C’ = 107, ‘gamma’ = 0.001, and kernel = radial basis function where ‘C’ is the regularization parameter and ‘gamma’ is the kernel coefficient; RF: ‘n_estimators’ = 1500 where ‘n_estimators’ is the number of trees in the forest. For training and testing the classifiers, we divided the data by 80% for training and 20% for testing at the subject level; the samples from 12 subjects were used for training and the samples from the other three subjects were used for testing (five-fold cross validation was adopted). We assumed that the five-fold cross validation appropriately divides the samples for the proper train and reliable test of the classifiers as well as the subject level separation to assess the effect of inter-subject variation in the hand gesture classification.
(5) For the performance evaluation, we calculated the accuracy, recall, precision, and F1 score of each classifiers. As well, confusion matrix and ROC curve were analyzed.

We used a laptop computer equipped with CPU (core i7-10510U@1.8GHz) and RAM of 16 GB for data processing, training classifiers, and analysis. We exploited scikit-learn (ver. 0.23.2), pandas (ver. 1.0.3), matplotlib (ver. 3.2.1), numpy (ver. 1.18.1) and other useful python libraries as well.

## 3. Results

**Figure 7:**
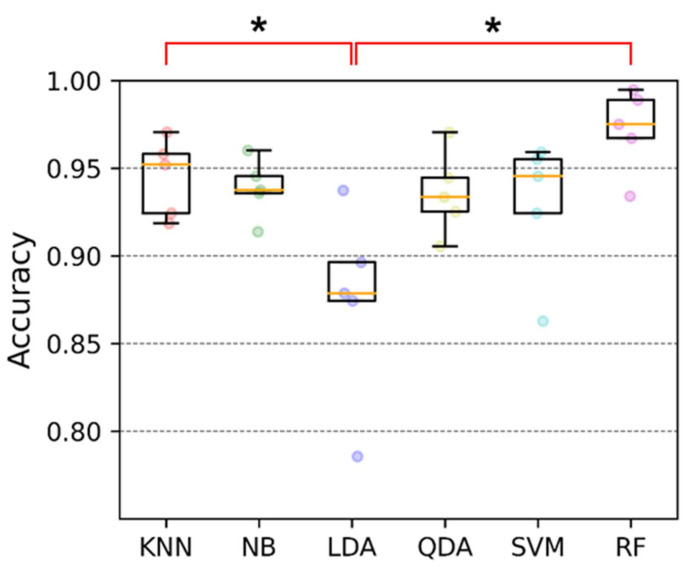
Accuracy of the classifiers; the median accuracies are KNN: 95.2%, NB: 93.8%, LDA: 87.9%, QDA: 93.3%, SVM: 94.6, and RF: 97.5%; the circles of each classifier express the accuracies of five-fold cross-validation; the orange line in the box plot shows median values. One-way ANOVA with Bonferroni post hoc test for the multiple comparison was performed. (*, *p* < 0.05).

**Figure 8:**
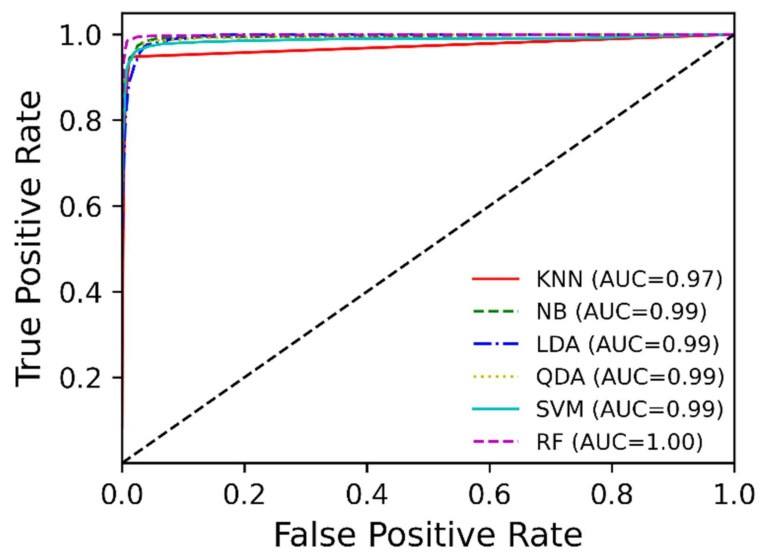
Receiver operating characteristic curves of the six classifiers; AUC and ROC curve were calculated by macro-average technique for the multi-class classification.

To estimate the performance in the hand gesture recognition via using the proposed soft sensors (the data glove), we investigated the accuracy, receiver operating characteristic curve, confusion matrix, recall, precision and F1 score. From the accuracy box plot as shown in Figure 7 and Table 2, the accuracy of LDA is 87.4% with ±5.6% standard deviation (SD). LDA shows the lowest accuracy and the largest SD among the 6 classifiers; KNN has 94.5% accuracy with ±2.2% SD, NB: 93.9% ± 1.7%, LDA: 87.4% ± 5.6%, QDA: 93.6% ± 2.4%, SVM: 92.9% ± 4.0%, and RF: 97.3% ± 2.4%. The best classification accuracy was established by RF and the smallest SD was by NB. The receiver operating characteristic curves of six classifiers are shown in Figure 8.

**Figure 9:**
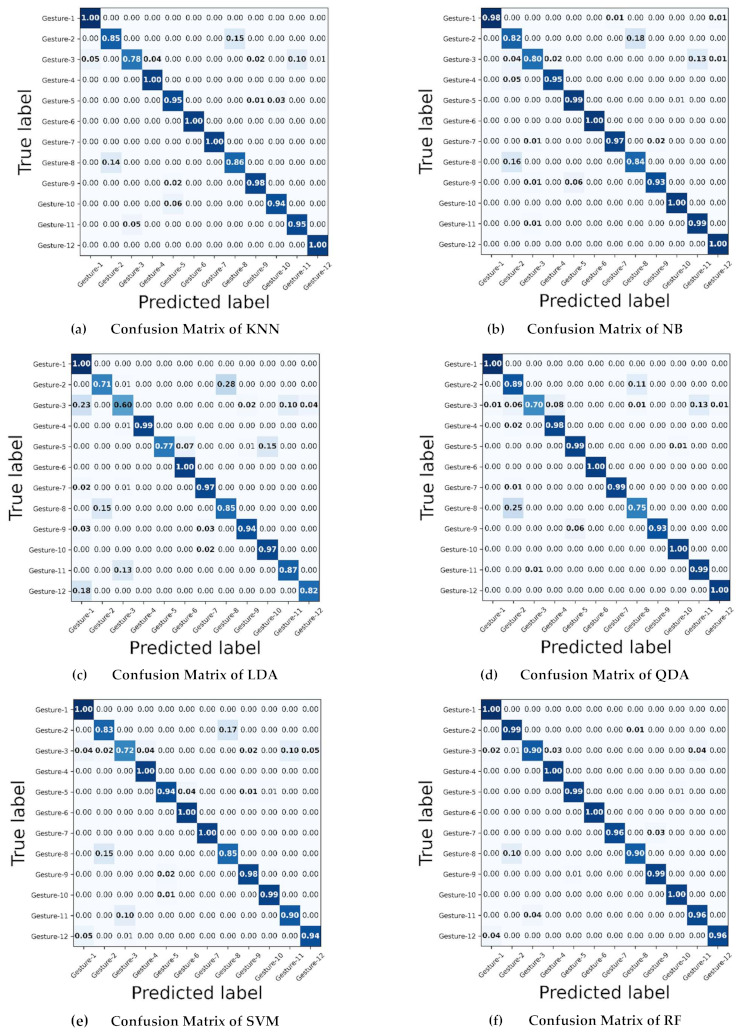
Confusion matrices of the classifiers; the total number of samples is 34,202 which was collected from 15 participants.

Figure 9 shows the confusion matrices of the six classifiers. The noticeable low classification accuracies, which means the classification accuracy of the individual gesture is less than 80% in the confusion matrices, are Gesture #3 (78%) in KNN; Gestures #2 (71%), #3 (60%), and #5 (77%) in LDA; Gestures #3 (70%) and #8 (75%) in QDA; and Gesture #3 (72%) in SVM. The values in the parenthesis are the classification accuracies of each gesture. Gesture #3 was less accurately recognized over the studied classifiers and it was mostly misclassified as Gesture #11, except that LDA mostly misclassified Gesture #3 as Gesture #1. This is a less-expected result; certainly, it is not enough to explain this result by only the similarity of these gestures, Gestures #3 and #11. It is because that there are other candidates excluding Gesture #11 with respects to the gesture’s shape similarity.

Another point is Gestures #2 and #8. All classifiers were likely to misclassify Gesture #2 as Gesture #8, vice versa. This misclassification is explainable by the gesture’s shape similarity; the only difference between these gestures is the posture of the thumb. Additionally, the misclassification of Gesture #5 in LDA is frequently Gesture #10; these gestures have only a difference of the position of the little finger. In RF, all classification accuracies of the individual gestures are over 80%.

**Figure 10:**
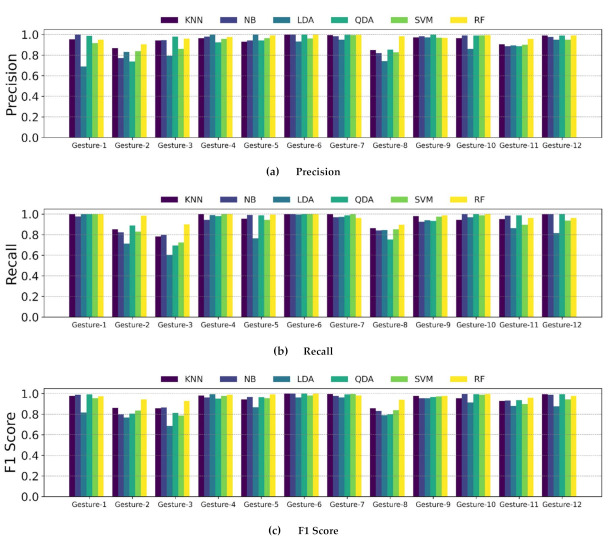
Precision, recall, and F1 score of the classifiers at each gesture; Gestures #2, #3, and #8 have relatively lower values than the other gestures; most of the cases shows that RF performs better than the other classifiers.

Figure 10 shows the recall, precision, and F1 score of each gesture and classifier. These three performance plots also show the similar tendency as presented in the results of the confusion matrix; the classification performance of Gestures #2, #3, and #8 are mostly lower than of the others over all classifiers.

## 4. Discussion

In this study, a fabric data glove was developed based on robust and highly flexible strain sensors using a silicone rubber embedding microchannels filled with a liquid metal EGaIn alloy. Moreover, the hand gesture recognition via the proposed glove was studied with the various classification techniques. The proposed soft EGaIn-silicone sensor was fabricated by rubber molding and casting and liquid metal injection methods. The EGaIn-silicone sensor exhibited some degree of nonlinearity and hysteresis characteristics, but showed a gradual increase in resistance as the applied uniaxial strain increases along to the longitudinal direction of the sensor. The preliminary test results also verified the repeatability of the strain measurement using the EGaIn-silicone sensor.

Additionally, as a possible application using the proposed glove, the hand gesture recognition was studied with the 12 static hand gestures. The performance of the glove in the gesture recognition with the traditional machine learning methods showed that high multi-class classification accuracy over 90%, except for LDA because of the non-linearity of the soft sensors. As shown in Table 3, this study has led to the comparable classification accuracy for hand gesture recognition via the developed data glove compared to other studies. The availability of the proposed data glove and soft sensors was confirmed from the results. However, this study also has a couple of limitations and we state the limitations in this section. Moreover, we discuss a couple of findings from the study as well. Details are as follows.

Gestures #2 vs. #8 and Gestures #3 vs. #11— From the confusion matrices in the Results section, it was found that Gesture #2 was frequently misclassified as Gesture #8. The difference between Gesture #2 and #8 is the position of the thumb; Gesture #2 is a fist motion with the bended thumb, but Gesture #8 is the thumbs-up motion which has the straightened thumb. This misclassification might come from the limitation of the proposed soft sensor; even though we installed the sensors on the palm side of the glove to increase their sensitivity, a few cases were noticed that the sensors were not able to detect the DoB of a bended finger. This was likely to be caused from the thickness of the sensor. With our restricted fabrication capability, reducing the thickness of the sensor was limited. The sensitivity of this thick sensor was imperfect to detect the bended finger. We speculate that this limitation also affects to the misclassification between Gestures #3 and #11.

LDA vs. QDA— For the results, LDA shows the worst classification accuracy. However, QDA shows relatively reasonable accuracy compared to the other classifiers. The key difference between LDA and QDA is the way of dealing with the covariance matrix of classes; LDA assumes that all classes have the same covariance matrix, but QDA assumes that each class has its own covariance matrix which means that each class has different variances [61]. We speculate that this difference helps to explain the non-linearity of the proposed soft sensor and QDA performed the hand gesture classification problem well; LDA did not. The other classifiers, KNN, NB, SVM, and RF, seem to handle the non-linearity of the senor adequately according to the results.

Limitations—This study has several limitations as follows.

First of all, the state-of-the-art deep learning techniques for the hand gesture classification were not investigated in this study. After the rapid growth of GPU-based machine learning techniques, i.e., deep learning, with a vast amount of data, numerous applications adopt the deep learning techniques to enhance the performance of their classification accuracy. However, the purpose of this study is to check the availability of the proposed data glove (or the EGaIn-silicone soft sensors) through the simply implementable algorithms, behaviors of which are easily interpretable as well. For the purpose, the traditional machine learning techniques used in the study were enough to evaluate the performance of the glove. However, for the further improvement in the gesture classification, should consider deep learning based classification approaches, e.g., one-dimension data based convolution neural network (1D CNN) [54], should be studied as well.

Moreover, we only used the steady state section of the gestures, which was visually identified, to evaluate the functionality of the proposed sensor as minimizing the effect of transient motions. However, this is not an applicable way to real-world applications. A future study needs to examine the transient motions as well as to recognize gesture sequences in real-time for practical applications. Additionally, we did not optimize the length of the sliding window, 200 ms, in this study. The length is perhaps able to affect the classification performance because the shorter sliding window can achieve the quicker response rate of the classification, but it is more vulnerable to motion artifacts and transient motions. This parameter is a tunable parameter to be optimized for practical applications. However, the results of the classification accuracy showed that 200 ms for the sliding window was an appropriate parameter for the reasonable classification performance. Surveying the effect of the parameter in the classification is slightly out of the topic of this study.

Another limitation is the fabrication capability to reduce the thickness of the sensor. The smaller cross-section of the EGaIn microchannel sensor causes the higher sensitivity. It means that the changes in resistance of the soft sensor increases as its microchannel cross-section decreases. We speculated that the misclassification of the specific gestures is likely to be alleviated by using the higher sensitivity sensor because we observed some cases where the finger were bent, but the voltage change of the soft sensor was not noticeable. To detect the voltage changes in the sensor, we used the simple voltage divider circuit with a relatively low resistance, but this was not an effective method because the current used in the circuit was slightly high. This high current issue can be resolved by using advanced voltage amplifier circuits in practical applications. In this study, we did not employ the amplifier circuits because our DAQ system was able to capture these low voltage changes in high resolution. However, this perspective should be considered so that it can be efficiently used in real applications.

Additionally, we did not independently account for the ability of measuring the pressure the sensor has in this study. The proposed sensor reacts to the pressure provided on the normal direction of the sensor and the stretching along the length direction as well. We used both abilities in a synthetic way to recognize the hand gestures. However, if these abilities are separately explained, the applicable fields using the proposed sensor may be broadened. A further study must consider this aspect of the proposed sensor.

Note that the proposed data glove did not take into account the measurements of the longitudinal forces in the palm generated during the stretching and squeezing motions of the palm for hand gestures. To integrate this component, additional sensors are required to be embedded to the palm area of the data glove. The relevant additional information is expected to help recognize a wider variety of hand gestures than the presented data glove. Using the advanced data glove to analyze the change in the palm side during hand gestures will be a valuable further study.

## 5. Conclusions

This study demonstrated the manufacturing procedure of EGaIn-silicone based soft sensors and developed a fabric data glove embedded with the proposed soft sensors. To evaluate the performance of the proposed data glove, 15 human subjects were recruited and 12 static hand gestures were tested. With the collected hand gesture dataset, the performance of the data glove in the hand gesture recognition was determined by the 6 traditional machine learning classifiers: K-Nearest Neighbors, Support Vector Machine, Linear Discriminant Analysis (LDA), Random Forest (RF), Naïve Bayes, and Quadratic Discriminant Analysis. LDA showed the lowest accuracy (87.4% ± 5.6%) and RF demonstrated the highest accuracy (97.3% ± 2.4%). The accuracy of LDA seems to be reduced due to the non-linear nature of the proposed soft microchannel sensor. However, the other traditional machine learning classifiers were able to overcome the non-linear characteristics of the sensor. This apparently states that a high computational deep learning technique is not needed to classify the hand gestures via the proposed sensors. Even though there still exists the performance degradation at classifying the similar gestures like Gesture #2 (hand close) and Gesture #8 (Num 5, thumbs-up), the recognition system using the proposed data glove shows sound classification accuracy for the rest of the hand gestures. In a future work, it is expected that the classification accuracy of a simple linear classification method can be improved by developing a closed-form equation that mathematically describes the non-linear behavior of the soft sensor.

## Figures and Tables

**Table 1:** Demographics of the study participants (mean+/−SD).

Age [yr]	Gender (M: Male, F: Female)	Height [cm]	Weight [kg]
32 ± 7	M: 13, F: 2	172.0 ± 7.6	71.4 ± 11.5

**Table 2:** Performance metrics of classifiers (mean ± SD).

Classifier	Accuracy	Precision	Recall	F1 Score
**KNN**	94.5% ± 2.2%	95.0% ± 2.0%	94.5% ± 2.2%	94.4% ± 2.3%
**NB**	93.9% ± 1.7%	94.4% ± 1.7%	93.9% ± 1.7%	93.8% ± 1.7%
**LDA**	87.4% ± 5.6%	89.5% ± 4.1%	87.4% ± 5.6%	87.1% ± 5.7%
**QDA**	93.6% ± 2.4%	94.3% ± 2.1%	93.6% ± 2.4%	93.4% ± 2.5%
**SVM**	92.9% ± 4.0%	93.5% ± 3.3%	92.9% ± 4.0%	92.7% ± 4.0%
**RF**	97.3% ± 2.4%	97.6% ± 1.9%	97.3% ± 2.4%	97.2% ± 2.4%

**Table 3:** Comparison of studies for a data glove based hand gesture recognition.

Reference	Sensor	Raw Data	# Gestures	# Users	Accuracy
Shukor et al. [55]	Tilt	10(tilt)	9	4	89%
Saggio et al. [56]	Flex(glove) + IMU(arm)	10 (flex) + 6 (IMU)	10	7	98%
Pezzuoli et al. [57]	Flex(glove) + IMU(arm)	10 (flex) + 2 (IMU)	27	5	99%
Huang et al. [58]	Reduced Graphene Oxide(RGO) coated fibers	10 (flex)	10	4	99%
Nassour et al. [44]	Potassium Iodide(KI)-Glycerol(Gly) + Conductive Liquid	14 (flex)	15	1	89%
Ciotti et al. [30]	Knitted Piezoresistive Fabrics	5 (stretch)	8	5	98%
Mummadi et al. [59]	IMU	5 (IMU)	22	57	92%
Wong et al. [60]	Capacitive	5 (capacitive)	26	10	99%
**This Study**	**EGaIn Microchannels**	**10 (stretch)**	**12**	**15**	**97%**

## Data Availability

The data presented in this study are available on request from the corresponding author.
